# Health Literacy, Social Support, and Kidney‐Specific Quality of Life Among Hemodialysis Patients in Saudi Arabia: Implications for Nurse Managers Organizing Patient and Family Education

**DOI:** 10.1155/jonm/9916107

**Published:** 2026-10-11

**Authors:** Romeo Jr Mostoles, Dolores Cabansag, Grace Ann Lim Lagura, Evalynn Rondilla, Eddieson Pasay-an, Reynita Saguban, Analita Gonzales, Nuha Ayad H. Alatawi, Jeneth Gutierrez

**Affiliations:** ^1^ College of Nursing University of Hail Ha’il Saudi Arabia uoh.edu.sa; ^2^ Department of Nursing Administration and Education College of Nursing King Khalid University Abha Abha Saudi Arabia kku.edu.sa; ^3^ Department of Nursing Administration and Education Faculty of Nursing University of Tabuk Tabuk Saudi Arabia ut.edu.sa; ^4^ Department of Medical Surgical Nursing Faculty of Nursing University of Tabuk Tabuk Saudi Arabia ut.edu.sa

**Keywords:** health literacy, nurse managers, nursing management, patient education, quality of life, renal dialysis, Saudi Arabia, social support

## Abstract

**Introduction:**

Limited health literacy and strong family involvement are both common among hemodialysis patients in Saudi Arabia, yet how each relates to kidney‐specific quality of life (KDCS), and what this implies for how dialysis units organize patient and family education, has not been examined. Decisions about education protocols, staff competencies, materials, timing, family inclusion, and quality monitoring are made by unit nurse managers, who currently lack local evidence to guide them.

**Aim:**

To examine the associations of health literacy and perceived social support with KDCS among hemodialysis patients in Hail, Saudi Arabia, and to identify what these associations imply for how nurse managers organize patient and family education in dialysis units.

**Methods:**

A cross‐sectional correlational design was employed with 215 patients recruited from two dialysis centers in Hail, Saudi Arabia. Health literacy, social support, and quality of life were measured using three validated Arabic‐language tools: the HLS‐EU‐Q47 (health literacy), the MSPSS (multidimensional social support), and the KDQOL–SF 1.3 (KDCS). Questionnaires were administered in a mixed mode: 81.9% (176/215) were interviewer‐administered following a standardized verbal protocol, and 18.1% were self‐completed with a researcher present. Descriptive statistics were calculated for all variables. Pearson correlations examined bivariate associations among the continuous scores. The primary inferential analysis was a multiple linear regression with continuous KDCS as the dependent variable and health literacy and social support as predictors, controlling for age and education. As a secondary, exploratory analysis, a chi‐square test examined the association between HLS‐EU‐Q47 health literacy categories and KDCS dichotomized at the sample mean.

**Results:**

We observed a pattern we descriptively term the “Support Paradox”: perceived family support was at the ceiling (*M* = 6.99/7.0, SD = 0.06), yet showed no association with KDCS (*r* = −0.09, *p* = 0.188; *β* = −0.08, *p* = 0.202). This null association may be explained by near‐ceiling family support scores and the resulting restricted variance, interviewer‐assisted administration (81.9% of surveys), and potential social desirability bias, and should be regarded as an observed pattern rather than evidence that family support is unrelated to quality of life. In the primary multiple linear regression model, health literacy was the only significant predictor (β = 0.19, *p* = 0.012), but the model explained less than 7% of KDCS variance (*R* ^2^ = 0.068, adjusted *R* ^2^ = 0.050), indicating a statistically significant but clinically modest association; 93.2% of KDCS variance remained unexplained by the model. In the exploratory categorical analysis, 91.7% of participants had limited health literacy, and sufficient health literacy was associated with “Good” KDCS (*X* ^2^ = 22.12, *p* < 0.001, Cramér’s *V* = 0.32). At the unit level, limited health literacy was near‐universal (91.7%), and most participants (81.9%), although literate, opted for verbal administration because completing written material during dialysis was impractical.

**Conclusion:**

Health literacy was a statistically significant but small contributor to KDCS, and perceived family support, although at the ceiling, was unrelated to it. Nearly all patients (91.7%) had limited health literacy, and most (81.9%) opted for verbal rather than written administration during dialysis, indicating that the response lies at the level of how the unit organizes education rather than with individual patients.

**Implications for Nursing Management:**

The findings define three responsibilities for nurse managers in dialysis units: organizing health literacy interventions, supporting culturally responsive patient and family education, and improving the quality of education. Because limited health literacy was near‐universal, managers should adopt a universal‐precautions approach in which plain‐language, verbal, and visual education with teach‐back is the default for every patient, supported by staff training and a standardized unit education protocol. Because families are consistently present and highly involved, managers should build culturally responsive, family‐inclusive education workflows that use Arabic‐language, visually based materials reflecting local food, religious, and family practices, and that direct teach‐back to the patient first to preserve patient autonomy and self‐management. Managers should monitor education quality through documented teach‐back, comprehension audits, caregiver attendance, and periodic reassessment of health literacy and QoL. Given the small effect size observed, these measures are necessary foundations whose impact on QoL must be evaluated prospectively.

## 1. Introduction

The global prevalence of chronic kidney disease (CKD) has reached approximately 9.1%. It accounts for over 1.2 million deaths annually [1]. In the Kingdom of Saudi Arabia, the burden is particularly significant. Initial studies indicate a 5.7% prevalence among younger populations [2]. For patients undergoing hemodialysis, disease management requires rigorous medication adherence, strict dietary and fluid restriction, and complex lifestyle adjustment [3, 4].

The study adopts the Sørensen et al. [5] integrated framework. This framework conceptualizes health literacy as the capacity to access, understand, appraise, and apply health information. These capacities inform decision‐making and self‐management behaviors. For hemodialysis patients, this pathway involves appraising complex clinical information (e.g., electrolyte balance and fluid targets) to guide dietary monitoring, fluid restriction, and treatment adherence, which in turn mitigates symptom burden and stabilizes quality of life (QoL). Within this framework, social support functions as a situational determinant. It facilitates health information appraisal. Thereby, it mitigates disease‐related burden [6].

The Sørensen model categorizes social support as a critical “situational determinant.” It may mediate the impact of health literacy on QoL. This aligns with the WHO perspective [7]. The WHO defines QoL as a subjective perception of one’s life position within a specific cultural and value system. Research emphasizes that in regions defined by strong social fabrics and familial interdependence, QoL is inherently tied to the efficacy of a patient’s social network in navigating illness. This suggests that for populations like those in the Hail Region, social support acts as a critical cultural buffer. They also mitigate the functional declines of advancing CKD [8, 9].

We hypothesize that in the Hail Region, the high density of familial support may coexist with, and possibly mask, deficits in individual health literacy, a pattern we refer to descriptively as a potential “Support Paradox.” We use this term descriptively to denote the co‐occurrence of these observations; it does not presuppose a causal mechanism, and we note in advance that a null support–QoL association may also arise from measurement factors such as ceiling effects. Under this hypothesis, near‐ceiling perceived family support would coexist with (a) poor kidney‐specific quality of life (KDCS); (b) no statistically significant correlation between support and QoL; and (c) reduced patient autonomous self‐management due to insufficient individual health literacy. This hypothesis is grounded in the Sørensen model. The model posits that situational determinants (e.g., social support) facilitate health information appraisal. However, if support operates primarily through emotional rather than instrumental channels, the expected positive association between support and QoL may be attenuated. The expected positive association between support and QoL may be attenuated or nullified, as it may be if family members assume decision‐making responsibilities. While international literature suggests general correlations between health literacy, social support, and QoL, the specific interplay remains undefined. This is especially true in a region where cultural values may shift the relative weight of individual literacy versus collective support [10]. Without empirical data from this specific sociocultural context, healthcare providers in Hail lack the evidence‐based strategies needed. They need these strategies to address the high symptom burden. They also need them to address the declining functional status reported in local hemodialysis units [11, 12].

The way hemodialysis patients receive health information is largely determined at the level of the dialysis unit. Unit nurse managers decide which education protocols are used, which staff competencies are developed and maintained, which written or visual materials are procured, when in the treatment session education is delivered, whether family caregivers are included, and which indicators of education quality are monitored [13]. Nurse managers’ influence on patient outcomes operates through exactly these organizational levers, namely staffing, competency development, standardized processes, and quality monitoring [14]. Where limited health literacy affects most of a patient population, health‐literacy frameworks recommend an organizational rather than an individual response: making clear communication the default for all patients rather than screening and targeting a few. Whether this applies to hemodialysis units in Hail and how family involvement should be structured within it requires local data on the distribution of health literacy, the nature of available social support, and their relationship with patients’ QoL. This study was designed to provide that evidence base for nurse managers.

This study examines the associations of health literacy and multidimensional social support with KDCS, each adjusted for the other and for age and education of health literacy and multidimensional social support with KDCS. The study focuses on patients undergoing hemodialysis in the Hail Region.

## 2. Methods

### 2.1. Study Design and Setting

A cross‐sectional correlational study was conducted from January through June 2025. It was conducted at two dialysis centers in Hail, Saudi Arabia (King Khalid Hospital and King Abdulaziz Dialysis Center). In accordance with the STROBE (Strengthening the Reporting of Observational Studies in Epidemiology) guidelines, we have detailed the study setting, participant eligibility, and measurement variables in the following subsections.

### 2.2. Participants and Sample Size

Participants were adults aged ≥ 18 years with CKD. They were receiving long‐term hemodialysis at the two study sites. Eligibility criteria included (1) mental competence to provide informed consent; (2) basic literacy (ability to read and understand simple Arabic text); and (3) willingness to participate. Basic literacy was required for two reasons: Participants needed to read the printed response cards and follow the items during administration, and the Health Literacy Survey European Questionnaire (HLS‐EU‐Q47) measures health literacy (accessing, understanding, appraising, and applying health information) rather than basic reading ability, so excluding nonreaders preserves the construct validity of the instrument. Literacy was confirmed at screening by trained researchers. The literacy criterion is distinct from the mode of administration, which is described under Data Collection. An a priori sample size calculation was performed using GPower 3.1.9.7 for multiple linear regression. The calculation used four predictors (age, education, health literacy, and social support), a moderate effect size (*f* ^2^ = 0.15), alpha =  0.05, and power = 0.80. The minimum required sample size was 85. A total of 215 participants were recruited. This yielded a post hoc power of 0.99.

### 2.3. Instrumentation and Cultural Adaptation

Data were collected using a four‐part structured questionnaire. All instruments utilized were previously validated Arabic versions. They had established reliability in Saudi populations. The questionnaire consisted of four sections: (1) sociodemographic data (age, gender, education, income, and clinical history); (2) the HLS‐EU‐Q47; (3) the Multidimensional Scale of Perceived Social Support (MSPSS); and (4) the Kidney Disease Quality of Life–Short Form (KDQOL–SF 1.3).

The HLS‐EU‐Q47 measures the ability to access, understand, appraise, and apply health information [15]. In this study, the Arabic version demonstrated high internal consistency (Cronbach’s alpha = 0.92).

The MSPSS is a 12‐item scale measuring perceived support from family, friends, and significant others [16]. The Arabic version’s reliability in this study was alpha = 0.88. In health outcome research, ceiling effects occur when scores cluster at the upper limit of a scale.

The KDQOL–SF 1.3 is an 80‐item instrument. It includes the generic SF‐36 and 11 kidney‐specific domains. Following standard RAND scoring guidelines, all items were transformed to a 0–100 scale. To maintain clinical relevance and statistical power, results are summarized into the Physical Component Summary (PCS), the Mental Component Summary (MCS), and the Kidney Disease Component Summary (KDCS). Higher scores on these summaries indicate better health‐related QoL [17].

### 2.4. Data Collection

Eligible participants were approached by the researchers at the dialysis session. After explaining the purpose of the study, the researchers obtained written informed consent prior to the surveys.

The questionnaires were designed for self‐administration but were delivered in a mixed mode. At the start of each session, participants were offered the choice of completing the questionnaire themselves or having it read aloud. Because the combined instrument comprised approximately 140 items and was completed during dialysis, with one arm immobilized and fatigue commonly reported, 176 participants (81.9%) chose verbal administration and 39 (18.1%) self‐completed with a researcher present to clarify terminology on request. All administration followed a written protocol. Researchers read each item and its full set of response options verbatim from the validated Arabic versions, without paraphrasing or interpretation; clarification was restricted to a pre‐agreed glossary; participants indicated their answers using a printed response card, and the researcher recorded the response. Administration took place in a designated quiet area of the unit, and family members or companions were asked to step away for the duration. All researchers received training in the protocol and completed supervised pilot administrations before data collection. Administration mode was recorded, but because the self‐completed subgroup was small (*n* = 39), mode was not entered as a covariate, and mode‐related differences in scores cannot be excluded. To reduce social desirability bias, researchers emphasized confidentiality and the use of coded identifiers before beginning and used neutral prompting throughout. Completed questionnaires were checked for missing entries before the participant left the session.

### 2.5. Ethical Considerations

The study received approval from the Institutional Review Board of the Hail Health Cluster (Log No. 2025‐92). All participants provided written informed consent. Participation was voluntary. Anonymity was maintained by using coded identifiers.

### 2.6. Statistical Analysis

KDQOL–SF 1.3 data were scored according to the RAND manual [17]. The KDCS was chosen as the dependent variable because it captures the burden specific to end‐stage renal disease and because a nonstandardized “Total QoL” sum would obscure the distinct physical, mental, and disease‐specific dimensions.

Analyses proceeded in three prespecified steps: Step 1 (descriptive): Means, standard deviations, and frequencies were computed for all sociodemographic, health literacy, social support, and KDQOL–SF 1.3 variables. Step 2 (bivariate): Pearson’s correlation coefficient (*r*) was used to examine linear relationships between the continuous HL Index, MSPSS scores, and KDCS, and a scatter plot with a fitted regression line was generated (Figure 1). Step 3 (primary inferential analysis): A multiple linear regression was conducted with the continuous KDCS score as the dependent variable and age, education level, HL Index, and MSPSS total entered simultaneously (enter method) as predictors. This model provided all effect‐size estimates (*B*, *β*,*R* ^2^, adjusted*R* ^2^, Cohen’s f^2^) reported in this study. Collinearity was assessed using the Tolerance and variance inflation factor (VIF).

**FIGURE 1:**
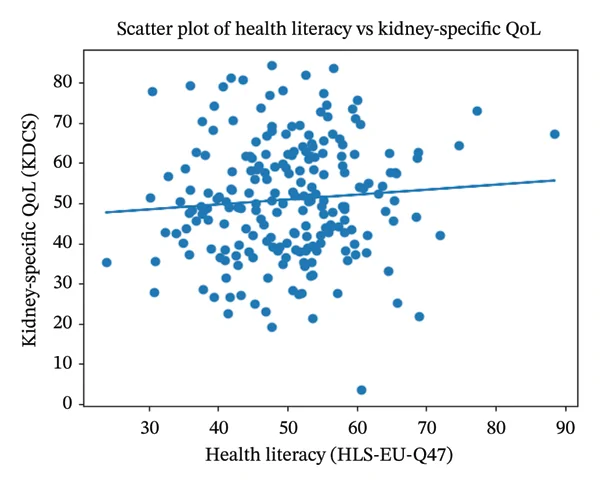
Scatter plot of health literacy and kidney‐specific quality of life.

Secondary, exploratory categorical analysis: To facilitate clinical interpretation, the HL Index was categorized using the published HLS‐EU thresholds (inadequate 0–25; problematic > 25–33; sufficient > 33–50), and KDCS was dichotomized into “Poor–Moderate” (≤ sample mean of 53.6) and “Good” (> sample mean). The association between these categories was examined with a chi‐square test and Cramér’s V (Table 1). KDCS was dichotomized because no clinically validated cut point for the KDCS exists in the literature; the sample mean was selected as a transparent, data‐driven threshold. Because dichotomization discards information and reduces statistical power, this analysis was treated as exploratory only and was not used for effect‐size estimation or hypothesis testing of the study aims. No logistic regression was performed; all inferential conclusions are drawn from the continuous multiple linear regression model (Table 2).

**TABLE 1:** Exploratory categorical analysis: association between health literacy levels and dichotomized kidney‐specific quality of life.

Health literacy level	Poor–moderate QoL	Good QoL	Total	*χ* ^2^	*p*
Inadequate	45	5	50	22.12	< 0.001
Problematic	108	39	147		
Sufficient	6	12	18		
Total	159	56	215		

*Note:* Degrees of freedom (df) = 2. Cramér’s V = 0.32 (medium by Cohen’s conventions; this value is inflated by dichotomization of KDCS and the small sufficient group, *n* = 18, and should not be interpreted as an estimate of effect magnitude; see Table 2).

**TABLE 2:** Multiple regression analysis predicting Kidney Disease Quality of Life.

Predictor	*β*	SE B	*β*	*t*	*p*
(Constant)	35.42	5.1		6.95	< 0.001
Age	−0.12	0.08	−0.11	−1.5	0.135
Education level	1.15	0.85	0.1	1.35	0.178
Health Literacy Index	0.28	0.11	0.19	2.55	0.012^∗^
Social support	−0.45	0.35	−0.08	−1.28	0.202
*R* ^2^ * = 0.068; F(4,210) = 3.82,* *p* = 0.005*.*					

*Note: N* = 215. Dependent variable: Kidney Disease Component Summary (KDCS) score. β = standardized regression coefficient. Model adjusted for age and education. *R* ^2^ = 0.068; adjusted *R* ^2^ = 0.05. CI = confidence interval. ^∗^p < .05 (two‐tailed).

## 3. Results

Table 3 presents the sociodemographics of the participants. The study included 215 hemodialysis patients. The cohort was predominantly over 30 years of age (92.6%), female (54.9%), and Saudi (87.9%). Socioeconomic indicators suggest a lower‐income profile, with 64.2% earning less than SAR 5000 monthly. Notably, nearly 69% of participants reported an unknown cause for their CKD.

**TABLE 3:** Sociodemographics of the participants.

Variable	Category	Count	(%)
Age	≤ 30 years	16	7.4
	> 30 years	199	92.6

Gender	Male	97	45.1
	Female	118	54.9

Marital status	Unmarried	72	33.5
	Married	143	66.5

Nationality	Saudi	189	87.9
	Non‐Saudi	26	12.1

Educational attainment	Primary	85	39.5
	Secondary	94	43.7
	Tertiary	36	16.7

Household income	< SAR 5000	138	64.2
	SAR 5001–10,000	39	18.1
	> SAR 10,001	7	3.3
	Others	31	14.4

CKD duration	≤ 3 years	72	33.5
	4–6 years	90	41.9
	≥ 7 years	53	24.7

Cause of CKD	Unknown	148	68.8
	HTN and diabetes	39	18.1
	HTN	24	11.2
	Other/glomerulonephritis	4	1.9

*Note: N* = 215.

Table 4 presents the descriptive statistics for QoL, health literacy, and social support. Participants showed moderate kidney‐specific adaptation (KDCS: 53.6) but poor physical (PCS: 19.4) and mental (MCS: 20.4) health. Health literacy was in the “Problematic” range (*M* = 32.85, SD = 7.2). Perceived family support was near‐ceiling (*M* = 6.99, SD = 0.06), yet showed no statistically significant correlation with KDCS (*r* = −0.09, *p* = 0.188), physical health, or mental health. We descriptively label this pattern the “Support Paradox”: high perceived emotional support coexisting with poor clinical well‐being and no statistical association. This null association should be interpreted cautiously. The near‐ceiling distribution (SD = 0.06) left almost no variance to correlate with KDCS, and 81.9% of questionnaires were interviewer‐administered, which may have inflated family support scores through social desirability bias.

**TABLE 4:** Descriptive statistics for QoL, health literacy, and social support.

Domain	Scoring metric	M	SD
KDQOL–SF 1.3 indices			
Physical Component Summary (PCS)	0–100 scale	19.4	6.7
Mental Component Summary (MCS)	0–100 scale	20.4	7.4
Kidney Disease Component Summary (KDCS)	0–100 scale	53.6	11.4
Health Literacy (HLS‐EU‐Q47)			
Standardized HL Index	25–90 index	32.85	7.2
Social Support (MSPSS)			
Family Support	1–7 Likert	6.99	0.06
Friends Support	1–7 Likert	6.07	0.32
Significant Others Support	1–7 Likert	6.73	0.27

*Note:* KDQOL–SF 1.3, Kidney Disease Quality of Life–Short Form; HLS‐EU‐Q47, European Health Literacy Survey Questionnaire. Scores for PCS, MCS, and the HL Index have been standardized to 0–100 and 25–90 scales, respectively, as per the original scoring manuals.

Abbreviation: MSPSS, Multidimensional Scale of Perceived Social Support.

Table 5 presents the health literacy classification. Nearly all participants (91.7%) possessed limited health literacy, with the vast majority falling into the “Inadequate” or “Problematic” categories.

**TABLE 5:** Health literacy classification.

Tool	Category	*n*	%
HLS‐EU‐Q47 HL Index	Inadequate (0–25)	50	23.3
	Problematic (> 25–33)	147	68.4
	Sufficient (> 33–50)	18	8.4

KDQOL–SF (KDCS)	Poor–moderate	159	74
	Good	56	26

*Note:* HLS‐EU‐Q47, European Health Literacy Survey Questionnaire. Percentages may not total 100% due to rounding. KDQOL–SF categories were created using the sample mean as the cut point (poor–moderate: ≤ sample mean; good: > sample mean). This approach was adopted for exploratory purposes; however, it lacks clinical validation and may not reflect clinically meaningful thresholds.

Abbreviations: KDQOL–SF, Kidney Disease Quality of Life–Short Form; KDCS, Kidney Disease Component Summary.

Table 1 presents the exploratory categorical analysis. A statistically significant association was found between health literacy categories and dichotomized KDCS, *χ* ^2^(2, *N* = 215) = 22.12, *p* < 0.001, Cramér’s *V* = 0.32. This cross‐tabulation is descriptive and exploratory; the primary inferential analysis and all effect‐size estimates are reported in the continuous multiple linear regression (Table 2).

Table 6 and Figure 1 present the correlation between health literacy, social support, and QoL. A small but statistically significant positive correlation (*r* = 0.176, *p* = 0.010; *r* ^2^ = 0.031) indicates that higher health literacy was associated with higher KDCS scores. Social support showed no significant relationship with QoL (*r* = −0.09, *p* = 0.188).

**TABLE 6:** Pearson correlation analysis (bivariate associations).

Variable pair	Pearson’s r	*p*
Health Literacy Index vs. KDCS (QoL)	0.176	0.010∗
Social Support (MSPSS) vs. KDCS (QoL)	−0.09	0.188
Health Literacy Index vs. Social Support (MSPSS)	0.12	0.079

*Note:* KDCS, Kidney Disease Component Summary. *p* < 0.05 is considered statistically significant.

Multiple linear regression analysis was conducted (Table 2) to quantify the association between health literacy and continuous KDCS scores while controlling for age, education level, and social support. The model was statistically significant [F (4, 210) = 3.82; *p* = 0.005], but explained only 6.8% of variance [*R* ^2^ = 0.068], indicating that the vast majority of QoL variance is indicating that the great majority of KDCS variance was not explained by the measured predictors. Only the Health Literacy Index reached statistical significance (β = 0.19, *p* = 0.012), a small standardized coefficient; each one‐point higher score on the HL Index was associated with a 0.28‐point higher KDCS score (*B* = 0.28, SE = 0.11). Neither education (*p* = 0.178) nor social support (β = −0.08, *p* = 0.202) was a significant predictors, consistent with the null bivariate association (*r* = −0.09, *p* = 0.188) and with the descriptive “Support Paradox” pattern, noting that restricted variance in the MSPSS limits the interpretability of this null result. The regression model used the continuous KDCS score as the dependent variable; categorical analyses (Table 1) are presented for descriptive comparison only and should not be interpreted as effect‐size estimates.

Collinearity diagnostics indicated no multicollinearity concerns. VIF values for age (1.03), education (1.09), health literacy (1.08), and social support (1.02) were well below the conventional threshold of 5.0, and tolerance values ranged from 0.92 to 0.98, all above the minimum threshold of 0.10. These values confirm that the predictors are sufficiently independent for reliable regression coefficient estimation.

## 4. Discussion

### 4.1. Perceived Support and the “Support Paradox”

Perceived family support was at the ceiling (*M* = 6.99, SD = 0.06), while physical (PCS = 19.4) and mental (MCS = 20.4) health scores were low, and social support was unrelated to KDCS in both bivariate (*r* = −0.09, *p* = 0.188) and multivariable (*β* = −0.08, *p* = 0.202) analyses. We use “Support Paradox” as a descriptive label for this pattern, not as a demonstrated mechanism. Two measurement explanations are sufficient to account for it. First, the ceiling effect left almost no variance in family support to covary with KDCS; under such a range restriction, a null correlation is the expected result and is not evidence that family support is unrelated to QoL [18, 19]. Second, 81.9% of questionnaires were interviewer‐administered. Despite a standardized protocol and confidentiality assurances, interviewer administration inflates socially desirable responses, particularly in collectivist settings where family reputation is valued [19–21], and may have contributed both to the ceiling effect and to attenuation of any true association. A third, substantive explanation is offered only as a hypothesis: The MSPSS measures perceived emotional rather than instrumental support [22], and families who provide strong emotional support may also assume decision‐making, reducing patient autonomy and self‐management [23]. This would be consistent with the Sørensen model, in which social support facilitates rather than replaces individual appraisal of health information [5]. Friends (SD = 0.32) and significant others (SD = 0.27) support were also range‐restricted and unrelated to KDCS.

### 4.2. Health Literacy and KDCS

Health literacy was the only significant predictor of KDCS, but the association was small by every metric: *r* = 0.176 (3.1% of variance), *β* = 0.19, and a full model explaining 6.8% of variance (adjusted *R* ^2^ = 0.050; *f* ^2^ = 0.073, a small effect [24]). Health literacy is therefore one small, statistically detectable contributor to KDCS in this sample, not a dominant determinant. The direction of the association is consistent with evidence that health literacy supports adherence and self‐management in chronic disease [25, 26]; its size is consistent with prior work in which health literacy, self‐efficacy, and self‐care together explained only 16% of quality‐of‐life variance in type 2 diabetes [27], and with evidence that health literacy effects are mediated by socioeconomic and contextual factors [28]. The exploratory categorical comparison (10.0% of patients with inadequate vs. 66.7% with sufficient health literacy in the “Good” KDCS category) rests on a small sufficient group (*n* = 18) and a sample‐derived cut point, and dichotomization exaggerates group differences relative to the continuous effect. Health literacy interventions are therefore a necessary but insufficient component of care; unmeasured factors such as comorbidity burden, dialysis adequacy, and symptom load are likely to account for much of the remaining variance.

### 4.3. Interpreting the Findings at the Unit‐Management Level

Three features of the results speak directly to the organization of education in dialysis units. First, limited health literacy was not a minority characteristic but the norm (91.7%; only 18 of 215 patients scored in the sufficient range). At this prevalence, screening individual patients to identify who needs simplified communication offers little; the more efficient management response is to make plain‐language, verbal, and visual education with teach‐back the standard of care for all patients, consistent with the health‐literacy universal precautions approach [29–31]. Second, family support was at the ceiling for almost every participant. Whatever its relationship to QoL, which in this sample was null and for which we have proposed only methodological and hypothesized explanations, family presence is a resource that already exists in the unit. Nurse managers can structure it, for example by scheduling education when caregivers are present and by designing sessions in which the caregiver supports, rather than substitutes for, the patient’s understanding. Third, 81.9% of participants, all of whom met a basic literacy criterion, accepted verbal administration because completing written material during dialysis was impractical. This suggests that written leaflets alone are an inadequate education medium in this setting and that education needs to be delivered verbally and visually, at a time and in a form that accommodates treatment‐related fatigue and an immobilized arm. These are decisions about staffing, protocols, materials, and scheduling, which fall within the nurse manager’s remit.

## 5. Implications for Nursing Management

Three features of the results bear directly on how education is organized in dialysis units, and each falls within the nurse manager’s remit over staffing, protocols, materials, and scheduling. First, limited health literacy was the norm (91.7%; only 18 of 215 patients scored in the sufficient range). At this prevalence, screening individual patients to identify who needs simplified communication offers little; the more efficient response is to make plain‐language, verbal, and visual education with teach‐back the standard for all patients, consistent with the health literacy universal precautions approach [29–31]. Second, family support was at the ceiling for almost every participant; whatever its relationship to QoL, family presence is an existing resource that managers can structure. Third, most participants (81.9%), although literate, opted for verbal administration because completing written material during dialysis was impractical, indicating that written leaflets alone are an inadequate education medium in this setting. Because health literacy explained only 6.8% of KDCS variance, the following actions should be regarded as necessary foundations for health‐literate dialysis care, to be implemented alongside attention to comorbidity, dialysis adequacy, and symptom burden, and evaluated prospectively.

### 5.1. Organizing Health Literacy Interventions

Nurse managers should (a) ensure that all dialysis nursing staff are trained in plain‐language communication and teach‐back, incorporate these skills into unit competency frameworks and annual appraisal, and maintain them through periodic refresher training; (b) adopt a standardized unit education protocol covering fluid and dietary restriction, medication, vascular access care, and symptom reporting, so that content and method do not depend on the individual nurse; (c) designate a health literacy champion in each unit to coordinate these activities; and (d) allocate protected time for verbal education within the treatment session, at a point that accommodates treatment‐related fatigue and an immobilized arm.

### 5.2. Supporting Culturally Responsive Patient and Family Education

Because family involvement is near‐universal in this population, education workflows should include family caregivers by default, scheduling sessions when they are present and giving them a defined role in reinforcing dietary, fluid, and medication instructions at home. At the same time, the protocol should safeguard patient autonomy: Questions and teach‐back should be addressed to the patient first, and caregivers asked to support rather than answer on the patient’s behalf. Materials should be in Arabic, use visual and pictorial formats validated with local patients, and reflect regional food, religious, and family practices. Digital visual aids may supplement, but should not replace, face‐to‐face verbal education.

### 5.3. Improving the Quality of Education in Dialysis Care

Managers should treat patient education as a monitored quality domain rather than an informal activity. Suggested indicators include the proportion of patients with documented teach‐back at defined intervals, periodic comprehension audits of key self‐management instructions, caregiver attendance at education sessions, and annual reassessment of health literacy (HLS‐EU‐Q47) and KDCS (KDQOL–SF 1.3). Results should feed back into staff training and protocol revision.

### 5.4. Study Limitations

The cross‐sectional design precludes causal inference, and the health literacy–KDCS association, although statistically significant, was small (*R* ^2^ = 0.068). Although all participants met the basic literacy criterion, 81.9% chose verbal administration because of the instrument’s length and the constraints of completing it during dialysis; despite a standardized protocol, this may have increased social desirability bias, particularly for family support items, and contributed to the ceiling effect on that subscale. The literacy criterion excluded patients unable to read simple Arabic text, so the true prevalence of limited health literacy in the wider Hail hemodialysis population is likely higher than the 91.7% observed. The MSPSS captures perceived emotional support but not instrumental support or support quality, so a cultural mechanism cannot be distinguished from a measurement artifact on these data. The KDCS categories in Tables 5 and 1 were derived from the sample mean and lack clinical validation. Finally, the findings come from two centers in one region and may not generalize to other parts of Saudi Arabia.

## 6. Conclusion

In this sample of hemodialysis patients in Hail, health literacy was a statistically significant but small contributor to KDCS, and perceived family support, although at the ceiling, was unrelated to it. The latter pattern is adequately explained by range restriction and administration mode; whether it also reflects a cultural dynamic in which families assume decision‐making remains a hypothesis. Future studies should use longitudinal designs, measures of instrumental support and patient autonomy, and instruments with wider response distributions and should test prospectively whether health literacy interventions improve QoL in this setting.

For nurse managers, the practical message is that limited health literacy is an attribute of the unit’s whole patient population, not of a subgroup to be screened for, and that written materials alone do not reach patients during dialysis. Plain‐language verbal and visual education with teach‐back should therefore be the default for every patient; family caregivers should be included by default while the patient remains the primary respondent, and education should be monitored as a quality domain. Given the modest amount of variance explained, these actions are best regarded as necessary foundations for health‐literate dialysis care whose effect on QoL should be evaluated prospectively rather than assumed.

## Author Contributions

RJM, EP, and GLL: conceptualized and wrote the draft of the study; DC, AG, and JG: analysis and interpretation of the data; SRS, ER, NAHA, and RS: data gathering and writing the manuscript; RJM and EP: wrote the final draft of the manuscript.

## Funding

This research has been funded by the Scientific Research Deanship at the University of Hail‐Saudi Arabia through project number RG‐23 214.

## Ethics Statement

Ethical approval was granted by the Institutional Review Board, Hail Health Cluster, Hail City, Kingdom of Saudi Arabia (IRB Log Number 2025‐92). The research team confirmed that all methods were performed in accordance with the relevant guidelines and regulations. Informed consent was obtained from all the subjects.

## Consent

Please see the Ethics Statement.

## Conflicts of Interest

The authors declare no conflicts of interest.

## Data Availability

The datasets used and/or analyzed during the current study are available from the corresponding author upon reasonable request.
